# Dual-energy micro-CT for quantifying the time-course and staining characteristics of *ex-vivo* animal organs treated with iodine- and gadolinium-based contrast agents

**DOI:** 10.1038/s41598-017-17064-z

**Published:** 2017-12-12

**Authors:** Juliana Martins de Souza e Silva, Julian Utsch, Melanie A. Kimm, Sebastian Allner, Michael F. Epple, Klaus Achterhold, Franz Pfeiffer

**Affiliations:** 1 Chair of Biomedical Physics, Department of Physics and Munich School of BioEngineering, Technical University of Munich, 85748 Garching, Germany; 2 Department of Diagnostic and Interventional Radiology, Klinikum rechts der Isar, Technical University of Munich, 81675 München, Germany; 3 Institute for Advanced Study, Technical University of Munich, 85748 Garching, Germany; 4 Present Address: Institute of Physics, Martin Luther University, Halle-Wittenberg, Germany

## Abstract

Chemical staining of soft-tissues can be used as a strategy to increase their low inherent contrast in X-ray absorption micro-computed tomography (micro-CT), allowing to obtain fast three-dimensional structural information of animal organs. Though some staining agents are commonly used in this context, little is known about the staining agents’ ability to stain specific types of tissues; the times necessary to provide a sufficient contrast; and the effect of staining solution in distorting the tissue. Here we contribute to studies of animal organs (mouse heart and lungs) using staining combined with dual-energy micro-CT (DECT). DECT was used in order to obtain an additional quantitative measure for the amount of staining agents within the sample in 3D maps. Our results show that the two staining solutions used in this work diffuse differently in the tissues studied, the staining times of some tens of minutes already produce high-quality micro-CT images and, at the concentrations applied in this work, the staining solutions tested do not cause relevant tissue distortions. While one staining solution provides images of the general morphology of the organs, the other reveals organs’ features in the order of a hundred micrometers.

## Introduction

Chemical staining of animal tissues is an appropriate strategy to increase the low inherent contrast of soft-tissues in X-ray absorption micro-computed tomography (micro-CT)^1–3^. With this strategy, three-dimensional (3D) structural information on mammals^2,4–6^ and other animals^3,7–9^ in their healthy or diseased states^10,11^ are easily accessed. Since the first works published about combining staining with micro-CT^10,12,13^, this strategy has supported several functional and morphological studies^5,12,14,15^.

Among the chemical agents explored for micro-CT, the list of those most often used includes a few medical contrast media applied in the clinic in Magnetic Resonance Imaging (MRI) and X-ray Computed Tomography (CT)^10,16,17^; and iodine (I_2_), either simply diluted in alcohol (ethanol or methanol) or in aqueous solution with potassium iodide (KI)^9,12,13,18^. Although those staining agents are widely used for micro-CT, still little is known about the staining times necessary to provide a satisfactory increase of contrast; the effect of the staining solution in creating tissue distortions and changes to the overall size of the sample; and the differences in the staining agents’ ability to stain specific types of tissues. Staining times varying from a few hours to some weeks are described in the literature^14,15^, sometimes making the protocols time consuming, thus, not applicable in laboratory routines. Although sample contraction due to the use of alcohol-based staining agents has already been described^9,18^, an evaluation of the benefits in contrast enhancement in the context of tissue shrinkage and distortions is still incomplete, as well as a comparison of the ability of a staining solution to stain one or other type of tissue.

The architecture of *ex-vivo* mouse heart and lungs has already been well described with staining combined with micro-CT^2,4,5,19–22^. Thus, rather than providing a morphological description of these organs, here we contribute to the studies of soft-tissues’ staining combined with micro-CT, by providing new quantitative information about the diffusion of staining solutions due to staining time, tissue type and staining solution type. For that, we stained *ex-vivo* mouse heart and lungs controlling the time and using two well known contrast-enhancing solutions: one based on iodine (I_2_ in ethanol, known as I2E) and the other on gadolinium (Gadovist). We imaged the samples with a dual-energy micro-CT (DECT) technique, exploring an easy-to-use image acquisition protocol based on the knowledge of the X-ray emission spectrum and the sample components. When compared to regular micro-CT, DECT has the great advantage of providing a quantitative description of the staining agent content within the organs. Thus, our measurements allow a segmentation of the staining agents within the tissues and we could then create 3D maps of the distribution of the contrast agents within the samples. Our results describe for the first time the major differences in contrast and in sample size due to staining time, tissue type and contrast agent. Furthermore, our measurements reveal that much shorter staining times than those usually described in the literature are enough to provide high-quality micro-CT images with high-resolution for these organs.

## Results

**Figure 1:**
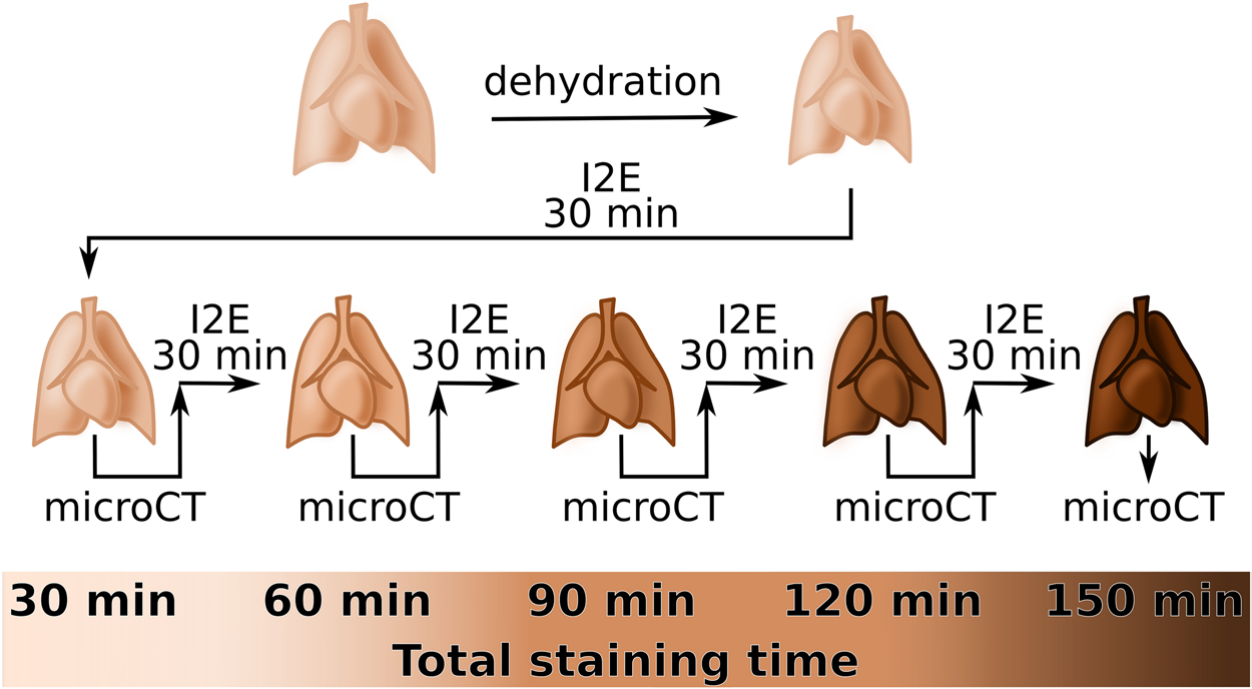
Workflow for the I2E staining procedure of the specimens. For I2E staining, the fixated samples were dehydrated in a graded series of ethanol solutions until 100 % ethanol (top row). Then, they were immersed in I2E staining solution for 30 min. Subsequently, the organs were imaged by dual-energy micro-CT, then the staining procedure was repeated for 30 more min, in a total of 60 min-staining. After micro-CT imaging of the organs stained for 60 min, the staining procedure was repeated, to a total of 90 min. The whole staining plus micro-CT imaging procedure was repeated using the same experimental parameter until the organs were stained for a total of 150 min. The colour gradient in the figures illustrates the increase staining solution accumulated in the organs along the staining time, that also cause a colour change observable with the naked eye. Similar procedure was performed with Gadovist for 60, 120 and 180 min staining-times and without dehydration step. Gadovist does not cause a change in the organs colour observable with the naked eye as suggested in the figure.

To study the effect of the staining time and the staining agent type on the contrast in micro-CT images, we performed sequential staining procedures on mice heart and attached lungs controlling the staining times and using either I2E or Gadovist as contrast-enhancing agent. We consecutively X-ray imaged the samples after each staining procedure, as illustrated in Fig. 1 for I2E staining. Imaging was performed with two different X-ray energy spectra, at a low- and a high-energy.

### Single-energy spectra imaging

**Figure 2:**
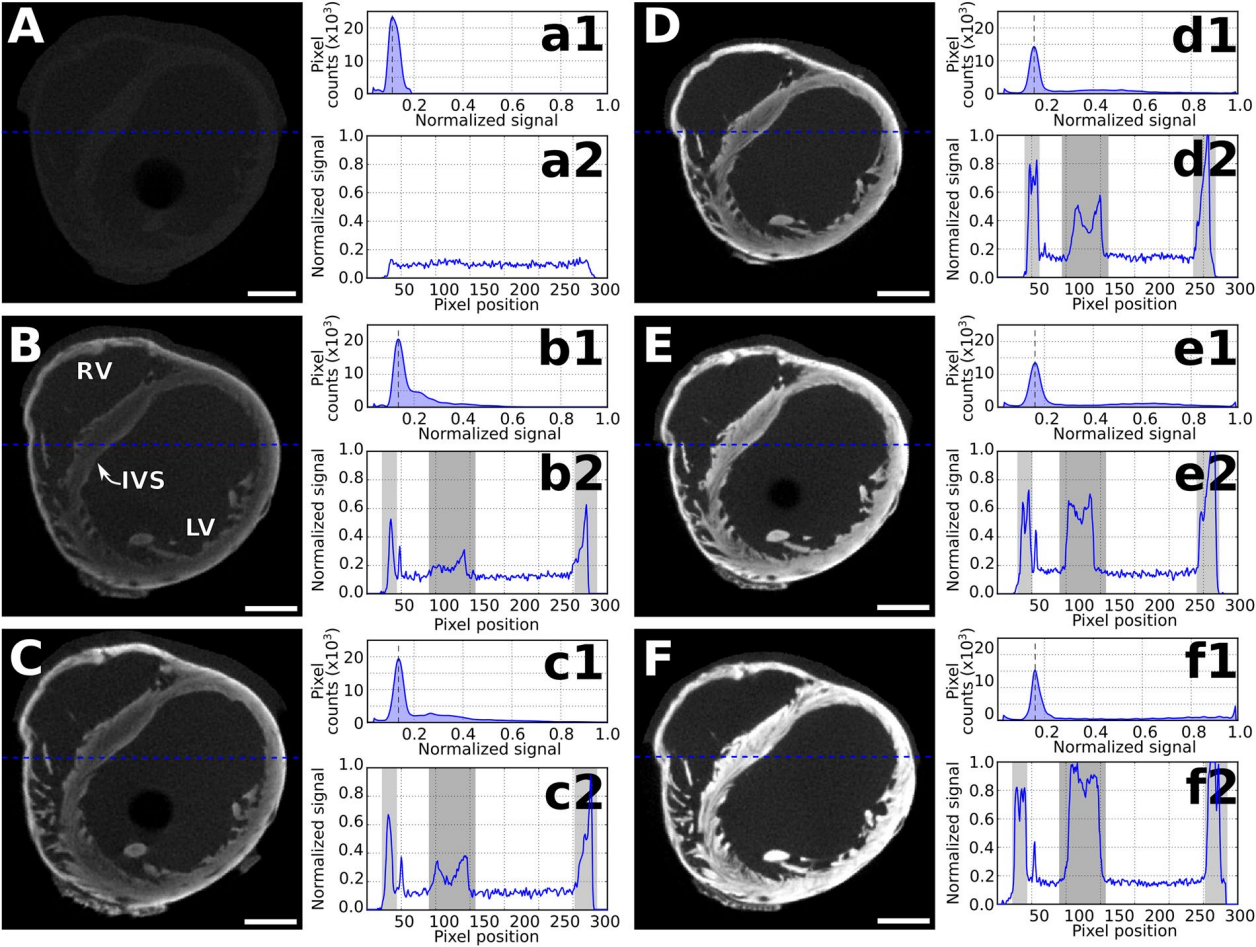
Comparison between X-ray micro-CT images (40 kVp) of non-stained and I2E-stained heart with increasing staining times. Tomographic slices of the heart are presented for staining times equal to (**A**) 0 min, (**B**) 30 min, (**C**) 60 min, (**D**) 90 min, (**E**) 120 min and (**F**) 150 min. The corresponding histograms (a1–f1) and line plots (a2–f2) are shown at the right side of each corresponding tomogram. Histograms show the pixel densities in the region of interest, with the peak on the left (black dashed line) representing the background and the peak on the right representing the soft-tissue. The IVS is the interventricular septum, LV the left ventricle, and RV the right ventricle. All grey values were normalised to the maximum signal presented in (**F**). Scale bar: 1000 μm.

A comparison between tomograms of non-stained and I2E-stained heart imaged at low-energy (40 kVp) shows that the non-stained sample delivers poor contrast among tissues in the organ and, thus, all sample features are lost in the low signal-to-noise ratio (Fig. 2A and a2). A better contrast is obtained in the tomogram of the heart stained for 30 min with I2E, with the borders of the organ well delineated (Fig. 2B). Indeed, the tomograms of the heart imaged in the same experimental conditions show an increase in contrast with the increasing I2E-staining times (Fig. 2A–F). This effect is clearly seen in the histograms, and after each staining round, the signal representing the gray values distribution of the sample continuously shifts away from the background signal (black dashed line in Fig. 2 and S1 a1-f1).

The details revealed with the staining time include the orientation of the muscular fibers in the intraventricular septum (IVS) and the inner and outer walls of the heart, which can be identified after only 60 min of staining (Fig. 2C). Though after 90 min the saturation of the staining agent in the tissue composing the heart walls is reached (Fig. 2 d2-f2, light-gray areas), longer staining times still cause an increase in the relative intensity of the signal related to the IVS (Fig. 2, a2–f2, dark gray area). Similar observations are also made for the lung tissue after each staining round with I2E, as a general increase in contrast is observed in the lungs, with the airways better delineated after each staining round (Supplementary Fig. S1). After 180 min of staining, I2E highlights structures of less than 100 μm in the lungs. Specifically in the esophagus, a more intense increase in contrast is observed compared with structures in the lungs (Supplementary Fig. S1, white arrow).

**Figure 3:**
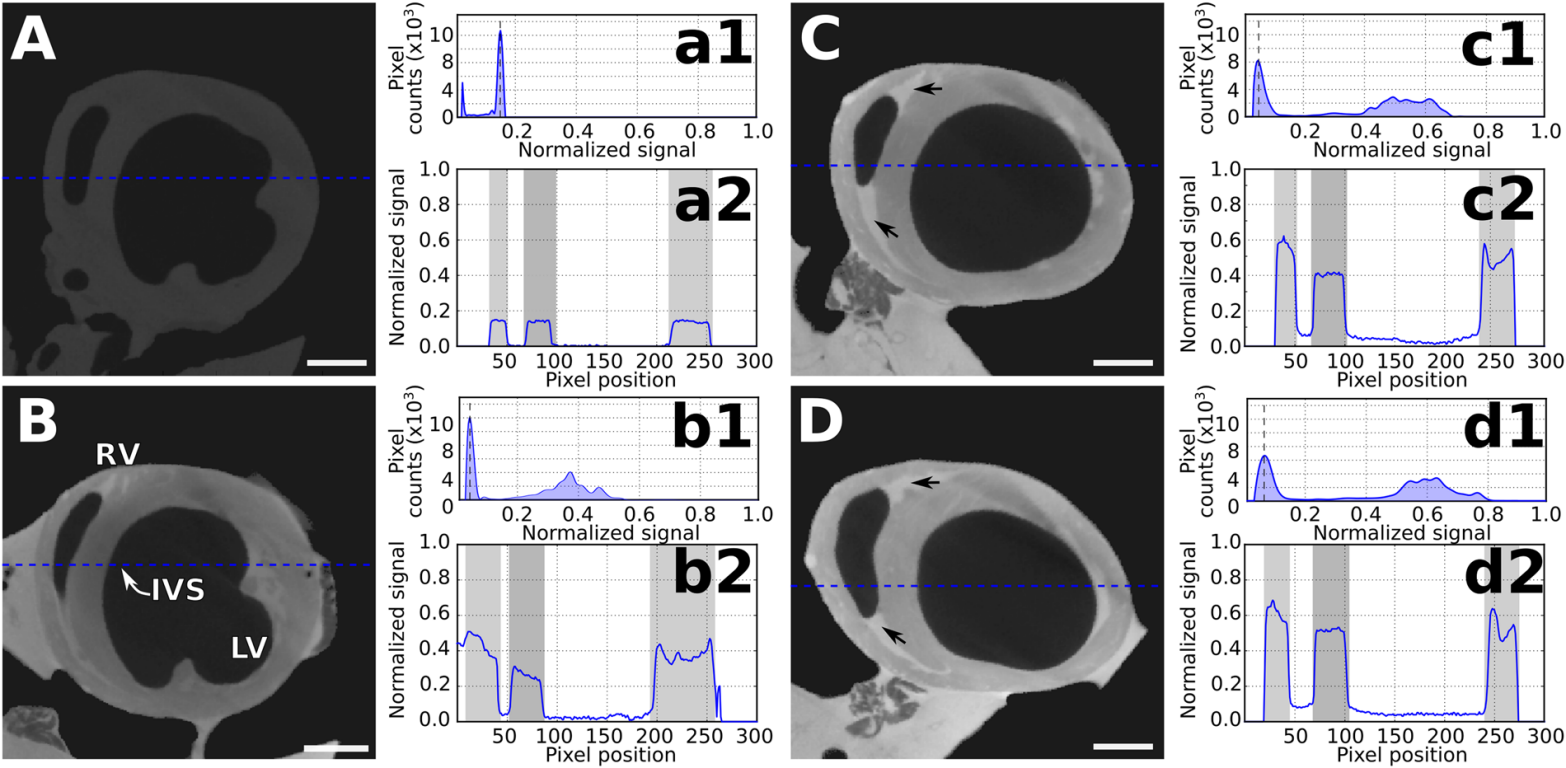
Comparison between X-ray micro-CT images (70 kVp) of non-stained and Gadovist-stained heart samples with increasing staining times. Tomographic slices of the heart are presented for staining times equal to (**A**) 0 min, (**B**) 60 min, (**C**) 120 min, (**D**) 180 min. The corresponding histograms (a1–d1) and line plots (a2–d2) are shown at the right side of each corresponding tomogram. Histograms show the pixel densities in the region shown in the respective tomogram, with the dashed black line indicating the peak related to the background and the signal on the right representing the soft-tissue. All grey values were normalised to the maximum signal presented in Fig. 2F. Scale bar: 1000 μm.

We performed a similar study with heart and attached lungs consecutively stained with Gadovist and imaged at a low energy (70 kVp). As observed for I2E staining, Gadovist-stained samples also show an improvement in contrast with increasing staining times, as seen in the tomograms, in the histograms and in the line plot values of the stained heart and lungs (Fig. 3 and S2). However, a general increase in contrast of the entire sample is seen after each Gadovist staining round, thus, almost no detail of the tissues’ morphology is revealed, such as the fibrous muscular tissue of the intraventricular septum and the heart walls or micrometric structures in the lungs. After the staining procedure, an amount of Gadovist solution remains in the heart ventricles (Fig. 3, black arrows). This same effect is observed in some lungs’ airways (Supplementary Fig. S2, black arrows), and the airways surface are then seen in a darker tone than the lung tissue and the airways.

**Figure 4:**
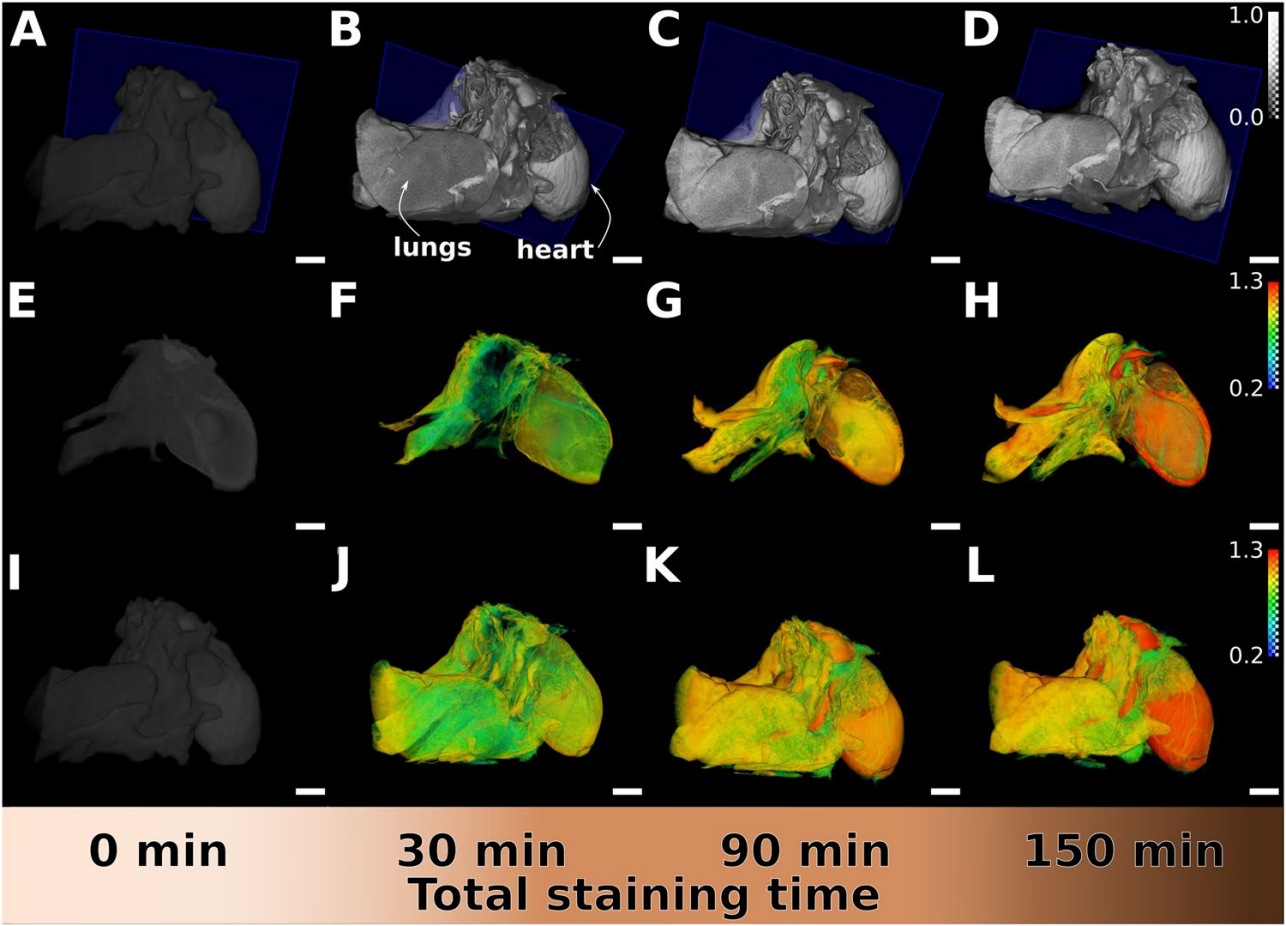
Volumetric renderings and iodine maps of non-stained and I2E-stained heart and lungs with increasing staining times. Samples are shown according to increasing staining times of 0, 30, 90 and 150 min, from left to right. Using the same gray values threshold, comparable tomographic slices of the non-stained and the stained organs are shown for the 40 kVp measurement (**A–D**). Iodine distribution in the organs was obtained after processing the data obtained with two different X-ray energy spectra. Iodine distribution is visible in the virtual cuts (**E–H**, corresponding to the blue planes in the images immediately above) and reconstructed volumes (**I–L**) of the heart and lungs with increasing staining times, with the red-coloured areas indicating the higher amount of iodine and green-coloured the lower, quantified with dual-energy experiments.

**Figure 5:**
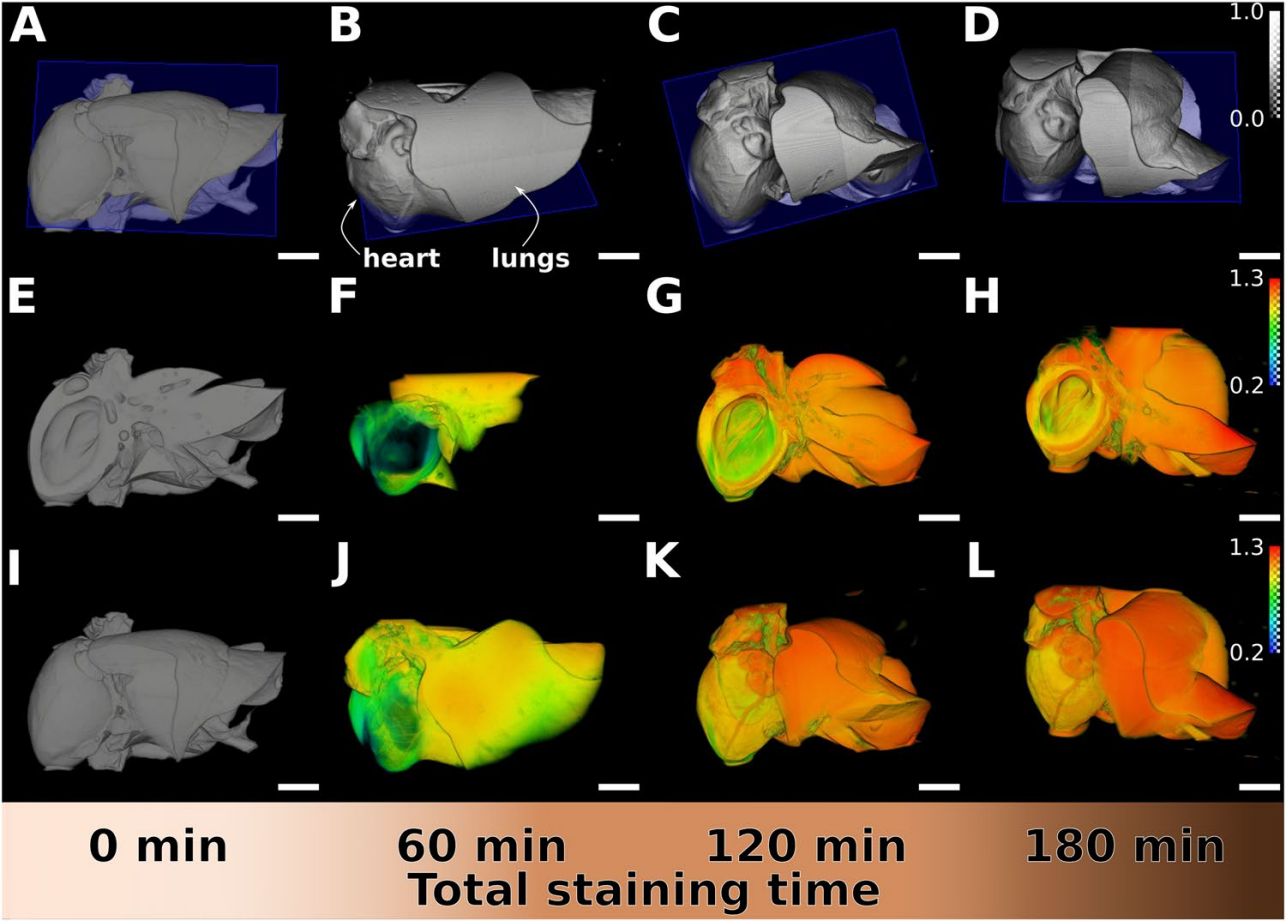
Volumetric renderings and gadolinium maps of non-stained and Gadovist-stained heart and lungs with increasing staining times. Samples are shown according to increasing staining times of 0, 60, 120 and 180 min, from left to right. Using the same gray values threshold, comparable tomographic slices of the non-stained and the stained organs are shown for the 70 kVp measurement (**A–D**). Gadolinium distribution in the organs was obtained after processing the data collected with two different X-ray energy spectra. Gadolinium distribution is seen in the virtual cuts of reconstructed volumes (**E–H**, corresponding to the blue planes in the images immediately above) and in the entire volumes of the organs (**I–L**) with increasing staining times. Red-coloured areas indicate the higher amount of gadolinium and green-coloured indicate the lower, quantified with dual-energy experiments. The sample is slightly more compressed onto the tube at 60 min and appears different in the first row.

The excellent contrast generated by longer staining times allowed us to better reconstruct the parts of the heart and lungs stained with I2E (Fig. 4A–D) and Gadovist (Fig. 5A–D).

### Dual-energy spectra imaging

Though the single-energy spectra imaging allows to observe the increase in contrast due to staining time, dual-energy micro-CT enables a deeper insight into the distribution of the staining media with a high spatial resolution. Dual-energy micro-CT quantifies the local concentration of the staining agent and we then used this technique to better identify the accumulation of the contrast agent on specific parts of the organs analysed. We processed the data obtained with two different X-ray energy spectra using an image-based material decomposition method for dual-energy X-ray computed tomography (Supplementary Fig. S3)^23^. This technique provides a quantification of the staining agent content regardless of the tissue density. Thus, in the maps of the distribution of iodine or gadolinium in the samples, we see the areas where the contrast-enhancing solutions tend to accumulate (Figs 4E–L and 5E–L, with the red-coloured areas containing the higher amounts, respectively, of iodine and gadolinium).

The concentration gradient of the contrast-enhancing element is clearly visualised from the outside to the inside in the sliced volumes of I2E-stained samples (Fig. 4F–H). According to the 3D staining map of iodine, the contrast is equally increased in the entire sample after the first staining round (Fig. 4F and J). After 90 min of staining, there is a differentiation of the lung tissue and the heart tissue, with the latter accumulating a higher amount of iodine (Fig. 4G and K). A 150-min staining time result in a more pronounced staining differentiation between heart and lung tissues, and the heart walls (Fig. 4H and L).

The gadolinium maps show that Gadovist accumulates mostly in the lungs than in other tissues after 60 min of staining (Fig. 5F and J). With longer staining times, this effect is pronounced and, after 180 min of staining, the profile of the lungs lobes is well delineated, the heart walls are depicted with the blood vessels highlighted on its surface (Fig. 5H and L).

**Figure 6:**
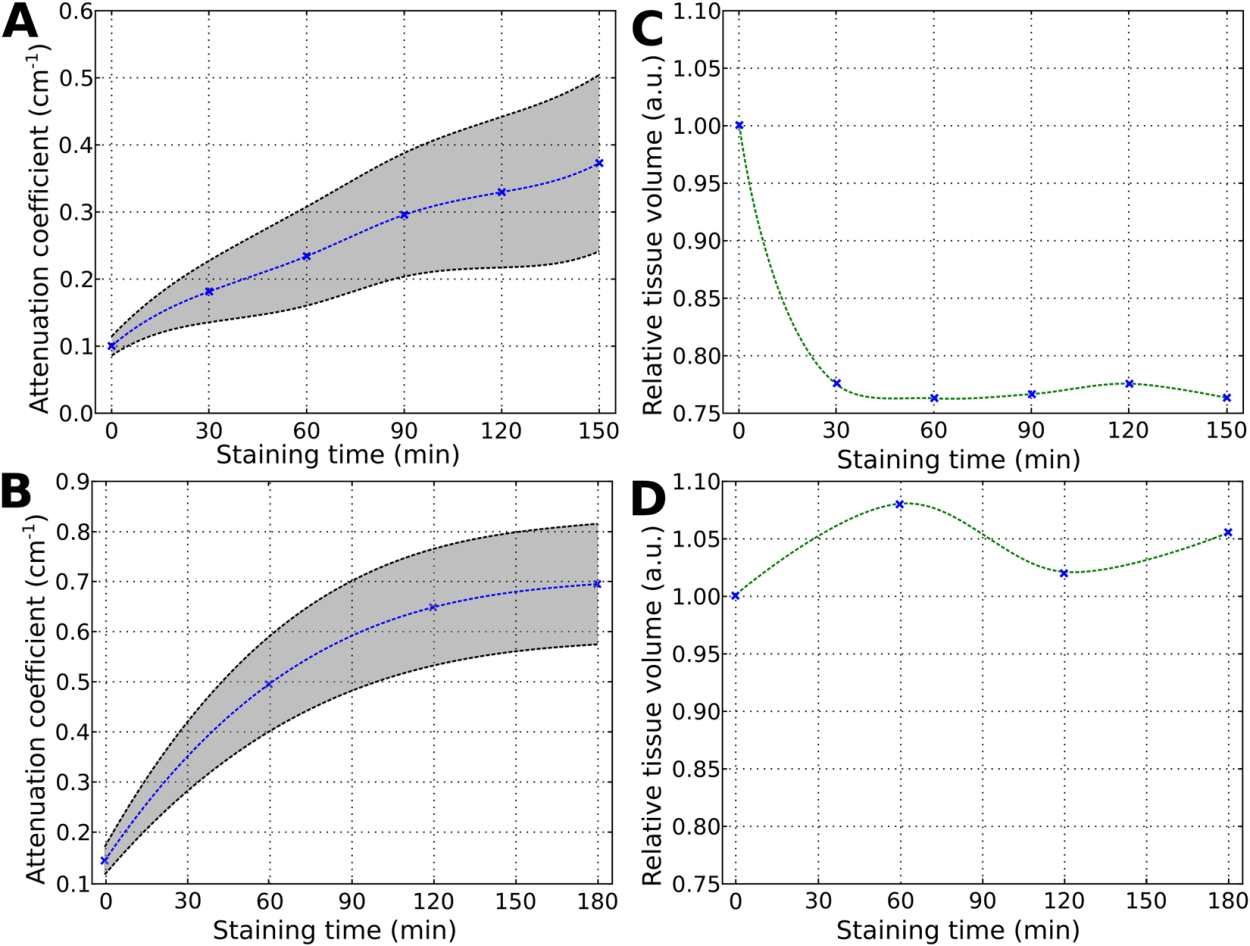
Samples’ relative volume and contrast changes with increasing staining times. For I2E staining, the increasing range of attenuation coefficient (average of sample within the grey area) along with the increasing staining times are seen in (**A**) and the changes in relative volume of the entire sample (heart and attached lungs) are seen in (**C**). Same data is shown for Gadovist staining in (**B**) and (**D**). The green and blue lines connecting the data points in (**C**) and (**D**) are not depicting a mathematical dependence and are only a guide to the eyes.

Due to longer staining times, along with the gain in contrast that can be quantified by the increasing range and values of attenuation coefficients (Fig. 6A and B), changes in the total samples volumes are also observed after the sequential staining procedures (Fig. 6C and D). A shrinkage of approximately 20% occurs after the first I2E-staining round, as expected after changing the organ from fixative aqueous solution to the ethanolic I2E solution. The sample size remains virtually unchanged after each of the further staining sessions (Fig. 6C). The sample size changes by less than 10% with the sequential Gadovist-staining procedures (Fig. 6D).

## Discussion

Our present study has focused on the time necessary for two commonly used staining solutions (I2E and Gadovist) to increase the contrast in micro-CT and on the differences observed in the accumulation of these contrast agents in the heart and lungs of *ex-vivo* mouse. We used an imaging protocol with two different X-ray energy spectra sequentially acquired. The single low X-ray energy spectrum tomograms and histograms of the samples stained with I2E (Fig. 2 and S1) and Gadovist (Fig. 3 and S2) show the uptake of these solutions in the heart and lungs, and the increase in contrast that is related to the staining agent and the staining time. Indeed, after each staining round, the gradual shift of the soft-tissue signal in the histograms is consistent with the wider range of intensity values of the samples stained for longer periods of time.

Though conventional micro-CT imaging (single-energy spectra) shows the increase in contrast due to staining time, the combined processing of the two datasets obtained with two different X-ray energy spectra result in a three-dimensional map of the staining agent content per voxel. This 3D map cannot be achieved with conventional micro-CT imaging because X-ray attenuation of the tissue cannot be eliminated even for a well-calibrated system. Dual-energy scans, on the other hand, allow quantitative calibration for the known absorbing materials (iodine and gadolinium) present in the staining agents used. Therefore here, to obtain a quantified distribution of contrast agent in the samples, we processed the two different X-ray energy spectra using an image-based material decomposition method for dual energy X-ray computed tomography (Supplementary Fig. S3)^24^. The main requirement for material decomposition on sequentially acquired computed tomographies is an adequate distinction of the X-ray energy spectra used with respect to the attenuating material for a broad-band X-ray source. This means that, either the spectra must differ sufficiently by being shaped with filters and different peak energies (Supplementary Figs S4 and S5), or that the attenuation coefficient of the main absorbing material varies significantly over the specified energy range. This is the case for the regime around an X-ray absorption edge. A combination of both was used for our choice of imaging parameters following to the obtained spectra. On one hand, this dual-energy imaging protocol helps discriminating two known materials in the measured sample as it allows differentiation of, for example, iodine or gadolinium from soft-tissue, independent of the density of the staining element and the total X-ray attenuation. On the other hand, it allows quantification of changes in concentration or density of a specific material over the entire sample with high sensitivity.

Image-based material segmentation methods suffer from image artifacts such as beam hardening, but the reliability of these techniques can be improved using the information obtained with material phantom measurements (Supplementary Figs S6 and S7). The material-selective volumes obtained by dual-energy imaging for iodine (Fig. 4) or gadolinium (Fig. 5) display the concentrations of these elements and allows quantification of the diffusion process, particularly in the three-dimensional view. The numbers on the colour bars in Figs 4 and 5 refer to the fraction of the iodine or gadolinium solution used for calibration of the setup. These solutions were identical to the media used for the staining (0.5% I_2_ w/v, named I2E; and pure Gadovist). In consequence, a comparison between the material-selective volumes for the different solutions shows that I2E first accumulates in the heart (up to approximately 1.3 times the staining solution concentration at longer staining times) and gives a strong contrast also for small sample features in the organ, whilst Gadovist has higher concentrations in the lung tissue (Fig. 5E–H and I–L) and does not depict the small details neither in the heart nor in the lung tissues. After 90 min, structural details are seen in the I2E stained heart, but not in the lungs. In contrast, the heart stained with Gadovist requires longer staining times than the lungs require to appear well depicted in the images. I2E increases the contrast of the heart walls more than the blood vessels (Fig. 5L) and Gadovist accumulates more in the blood vessels and less in the heart walls, thus, thus highlighting the heart blood vessels. Another difference consists in the areas where the staining solution accumulates in the lungs and adjacent tissues: I2E accumulates in the airways walls of the lungs (bronchial tree) and in the esophagus (Supplementary Fig. S1, arrow), while the Gadovist diffuse and accumulate in the entire organ, but not much in the esophagus and in the airways walls, that therefore appear in a shade (Supplementary Fig. S2).

The general differences observed between I2E and Gadovist staining are related to their ability to stain the heart, the esophagus and lungs’ airways. Our results agree with published data, which show that among other types of soft-tissues, the iodine-stained muscular tissue shows the highest grayscale values when compared to other types of tissues^9^. Indeed, according to the literature, muscle tissues (like those composing the heart) and epithelial tissues (lining the esophagus and parts of the bronchial tree) are well stained by iodine-containing solutions (both water- and ethanol-based) possibly due to local anisotropy in tissue diffusivity^9^. For Gadovist staining, our results suggest that the diffusibility of Gadovist into the lungs’ airways plays a more critical role than the local anisotropy of muscular tissue of the heart. The difference in the staining process between I2E and Gadovist is also illustrated by the different profile of the curves showing how the values of attenuation coefficients change with the staining time (Fig. 6A and B).

Our results show that I2E staining caused a more pronounced change in size than Gadovist staining (Fig. 6C and D). Sample shrinkage is already described in the literature for I2E^9,18^, due to the presence of ethanol used as solvent, which is known to remove water from tissues^9,25^. Also, the I2E staining procedure used here involved a previous step of dehydration of the organs in ethanol, that is known to increase cells membrane permeation, helping iodine diffusion into the organs^2^. Thus, the overall size differences observed between I2E- and Gadovist-stained samples are more likely to be due to the dehydration effect of the ethanol used in the I2E staining solution. Still, the dehydration caused by ethanol does not seem to cause distortions in the heart and lung tissue, at least in the sample features that are observable in the images presented here. In contrast, the Gadovist is a water-based solution^26^, that causes virtually no sample shrinkage. The variations in volume observed for Gadovist, and for I2E after 30 min of staining, could be due to small errors in the automated size measurements or due to small changes in the position of the sample in the plastic tube during the staining steps.

Besides observing the staining maps, another way of interpreting dual-energy CT results is the use of two-dimensional correlation histograms, which represent the number of voxels with a certain combination of effective polychromatic attenuation coefficients measured for low- and high-energy (Supplementary Fig. S8 for I2E-stained sample and Supplementary Fig. S9 for Gadovist-stained sample)^27^. The two-dimensional correlation histograms results show that, whilst only a small range of attenuation coefficients represent soft-tissue (organ), plastic (sample container) and air (negative values correspond to scattering by the sample), the diversity of attenuation coefficients increases rapidly due to the staining. With increasing staining times, the number of voxels with a large attenuation coefficient corresponding to a high amount of I2E also increases, which refers to iodine accumulation in the tissue (Supplementary Fig. S8). In the correlation histograms for staining with Gadovist, the amount of the staining solution in the lung tissue is clearly differentiated from the amount in the heart tissue (Supplementary Fig. S9). Indeed, over time the lung tissue becomes saturated and the Gadovist concentration inside the heart approaches saturation at the longest staining time used in this work.

In conclusion, our easy-to-use dual-energy X-ray micro-CT acquisition protocol and data analysis allow the quantification and the segmentation of staining agents within soft-tissues samples. Our results describe the major differences in contrast due to the staining time, the staining agent used and the organ studied. Supported by the 3D mapping of the contrast agent accumulation in the organs, our results show a clear improvement in contrast after every staining round with the two staining solutions tested, which are both commonly employed in micro-CT. Moreover, they show that the staining solutions used here diffuse differently in heart and lung tissues, and the saturation of some tissues is virtually reached in less than 3 h of staining procedure. Thus, we show that much shorter staining times than those described in the literature (ranging from hours to weeks) are enough to generate contrast to provide high-quality micro-CT images with high-resolution. When comparing the two staining solutions used, Gadovist is a good contrast agent to reveal the general morphology of the organs studied, while I2E provides more detailed images with features in the order of a hundred micrometers, though heart’s blood vessels are well depicted by both staining solutions. Also, the combination of two effects, the local anisotropy in tissue diffusivity and the higher permeability of tissues immersed in ethanol solutions, are in agreement with I2E being capable of staining the heart, the esophagus and the bronchial tree faster than it stains the lungs tissues, while the opposite is observed for Gadovist.

## Methods

### Samples preparation

Organ removal was approved from an internal animal protection committee of the preclinical centre (ZPF) of Klinikum rechts der Isar, Munich, Germany (internal reference number 4-005-09). Two 6 months old female C57Bl/6 mice (Charles River Laboratories, Europe) were sacrificed in accordance with relevant guidelines and regulations, and lungs and hearts were excised and fixed in 1% formaldehyde/ 2,5% glutaraldehyde in 1x PBS.

All staining and imaging procedures were performed in the same container, and care was taken to prevent the samples moving in between staining procedures to avoid alignment issues during image processing. Only freshly prepared staining solutions were used in all staining steps. For micro-CT imaging of the staining time 0, the sample was removed from the fixative solution and inserted in a plastic tube with a small volume of fixative solution in the bottom of the tube, not touching the sample. Imaging was performed with the tube closed to prevent the sample from drying. Immediately afterwards, the heart and attached lungs were dehydrated in a graded series of ethanol solutions (50, 70, 80, 90, 96% and absolute ethanol for 1 h each), then they were left in the iodine staining solution (0.5% w/v I_2_ in absolute ethanol, named I2E). After 30 min-staining, the staining solution was removed from the container, the organs were washed 3 times in pure ethanol and the sample was imaged in a closed plastic tube containing enough ethanol to prevent the sample from drying, but without touching it. This 30 min staining procedure followed by micro-CT imaging was repeated consecutively 5 times, thus, the organs were imaged for staining times equal to 30, 60, 90, 120 and 150 min.

For the heart and lungs stained with Gadovist™ (Bayer Healthcare - solution for intravenous injection containing 604.72 mg mL^−1^ of Gadobutrol (1.0 mmol mL^−1^ solution of 10-(2,3-dihydroxy-1-hydroxymethylpropyl)-1,4,7,10-tetraazacyclododecane-1,4,7-triacetic acid)), a similar procedure of staining followed by micro-CT imaging was performed. Though, since both Gadovist and fixative solutions are both aqueous solutions, the dehydration step necessary for I2E staining was not performed here. Heart and lungs were removed from the fixative solution and first imaged with micro-CT, corresponding to the staining time 0. Then, they were washed 3 times in deionised water and stained for 60 min with pure Gadovist. This 60 min staining procedure followed by micro-CT imaging was repeated consecutively 2 more times, thus, the organs were imaged for staining times equal to 60, 120 and 180 min.

### Data acquisition

Due to the possibility to maintain a sub-micrometer-resolution even for large samples, a VersaXRM-500 3D X-ray microscope from Zeiss was used to acquire the X-ray computed tomography measurements. All measurement parameters were chosen in a way to reach an appropriate signal-to-noise ratio for a sufficiently short scan time, to avoid sample movement during the scan. A dual energy technique was used to attain an iodine or gadolinium-specific image, which particularises the element distribution over the heart and lungs samples. The dual energy technique relies on the energy dependence of the X-ray attenuation for different materials (Supplementary Figs S6B and S7B). The samples were imaged with a low energy spectrum (40.2 kVp for the iodine-stained sample and 60.2 kVp for the gadolinium-stained sample) and immediately after with a high energy spectrum (70.1 kVp for the iodine-stained sample and 140.0 kVp for the gadolinium-stained sample), using the parameters in Table S1. Additionally, filter materials supported a sufficient separation of the X-ray emission spectra observed for every measurement (Supplementary Figs S6A and S7A).

For the calibration of the system, phantoms consisting of a rod of PMMA as a soft-tissue equivalent material and an Eppendorf tube filled with the staining agent 0.5% I2E or Gadovist™ were used (Supplementary Figs S4A,B and S5A,B). They were measured with the same scanning parameters as for the main measurements (Supplementary Table S1) and the material decomposition algorithm was tested on them (Supplementary Figs S4C,D and S5C,D). The attenuation coefficients of the basis materials were calculated as the mean values of the reconstructed PMMA rod and the content of the Eppendorf tube (Supplementary Table S2).

### Digital image processing and dual-energy decomposition

The image processing steps are presented in the flowchart in Supplementary Fig. S3 and described in the following text. To achieve high accuracy in image registration, it was necessary to align the dual-energy datasets with each other before reconstruction and to perform an additional post-reconstruction image alignment of the volumes. These steps consider a shift in both translation and rotation. Furthermore, median filtering was used for noise reduction. The sample segmentation was done by drawing region of interests manually and performing linear interpolation in between.

The dual-energy decomposition makes use of two CT scans consecutively acquired at two different energy spectra (Supplementary Figs S8A and S9A) to isolate the contribution of a specific material. Especially for the non-simultaneous scanning procedure, which does not require a designated dual-energy CT setup, it is recommended to perform the decomposition post-reconstruction as this simplifies the necessary image registration significantly. Even though this method does not prevent image artifacts such as beam hardening, it provides a satisfying quantitative description of the material contents, which gives an insight into the distribution of a contrast medium and enhances the understanding of the molecular behaviour.

For material decomposition we used a linear optimisation approach with non-negative constraints^23^. This solves voxel-wise systems of equations describing a composition of two materials given by:

$$\begin{array}{c}{\mu }_{{\rm{HE}}}({\rm{x}},{\rm{y}},{\rm{z}})={\mu }_{\mathrm{stain},\mathrm{HE}}\ast {{\rm{F}}}_{{\rm{stain}}}({\rm{x}},{\rm{y}},{\rm{z}})+{\mu }_{\mathrm{PMMA},\mathrm{HE}}\ast {{\rm{F}}}_{{\rm{PMMA}}}({\rm{x}},{\rm{y}},{\rm{z}}),\\ {{\rm{\mu }}}_{{\rm{LE}}}({\rm{x}},{\rm{y}},{\rm{z}})={\mu }_{{\rm{stain}},{\rm{LE}}}\ast {{\rm{F}}}_{{\rm{stain}}}(x,y,z)+{\mu }_{\mathrm{PMMA},\mathrm{LE}}\ast {{\rm{F}}}_{{\rm{PMMA}}}({\rm{x}},{\rm{y}},{\rm{z}}).\end{array}$$

The polychromatic attenuation coefficients µ_HE_ (high energy) and µ_LE_ (low energy) are given by the flat field corrected reconstructed volumes. The values µ_stain_ and µ_PMMA_ can be obtained from calibration measurements as explained above. The resulting material-specific F(x,y,z) gives the fraction of each calibration material for every voxel. Using air as a third material for the decomposition leads to an improvement of the results. The attenuation coefficients are given as the mean value of phantom-free regions in the reconstructed calibration measurements. The systems of equations change to:

$$\begin{array}{c}{\mu }_{{\rm{HE}}}({\rm{x}},{\rm{y}},{\rm{z}})={\mu }_{\mathrm{stain},\mathrm{HE}}\ast {{\rm{F}}}_{{\rm{stain}}}({\rm{x}},{\rm{y}},{\rm{z}})+{\mu }_{\mathrm{PMMA},\mathrm{HE}}\ast {{\rm{F}}}_{{\rm{PMMA}}}({\rm{x}},{\rm{y}},{\rm{z}}){+\mu }_{\mathrm{air},\mathrm{HE}}\ast {{\rm{F}}}_{{\rm{air}}}({\rm{x}},{\rm{y}},{\rm{z}}),\\ {\mu }_{{\rm{LE}}}({\rm{x}},{\rm{y}},{\rm{z}}){=\mu }_{\mathrm{stain},\mathrm{LE}}\ast {{\rm{F}}}_{{\rm{stain}}}({\rm{x}},{\rm{y}},{\rm{z}}){+\mu }_{\mathrm{PMMA},\mathrm{LE}}\ast {{\rm{F}}}_{{\rm{PMMA}}}({\rm{x}},{\rm{y}},{\rm{z}}){+\mu }_{\mathrm{air},\mathrm{LE}}\ast {{\rm{F}}}_{{\rm{air}}}({\rm{x}},{\rm{y}},{\rm{z}}),\\ \quad \quad \quad \quad {1={\rm{F}}}_{{\rm{stain}}}({\rm{x}},{\rm{y}},{\rm{z}}){+{\rm{F}}}_{{\rm{PMMA}}}({\rm{x}},{\rm{y}},{\rm{z}}){+{\rm{F}}}_{{\rm{air}}}({\rm{x}},{\rm{y}},{\rm{z}}){\rm{.}}\end{array}$$

The validation of the algorithm is shown by the decomposition of the phantoms used for calibration (Supplementary Figs S4 and S5).

### Sample volume evaluation

The volume of the samples was computed from the low energy CT data. The sample container was removed manually by a drawing tool in FEI Avizo Fire 8.1 and voxels consisting of air were set to zero by simple thresholding. Holes and chambers inside the sample were excluded from this step and they also contribute to the sample total volume. The whole number of voxels containing values larger than zero were counted automatically and multiplied with (pixel size)^3^ to obtain the final volume (see Table). Though the size measurement approach might not give an absolute size value, it is an accessible method to compare sample volumes changes.

### Availability of data and material

The datasets used and/or analysed during the current study are available from the corresponding author on reasonable request.

## Electronic supplementary material

Supplementary Information
