# Supplementary material for: Dual-energy micro-CT for quantifying the time-course and staining characteristics of ex-vivo animal organs treated with iodine- and gadolinium-based contrast agents
Source: Sci Rep. 2017 Dec 12;7:17387. doi: 10.1038/s41598-017-17064-z (PMC5727238; doi:10.1038/s41598-017-17064-z)
Supplement: Supplementary file 1 — Supplementary Information [file 41598_2017_17064_MOESM1_ESM.pdf]

Supplementary information:

Dual-energy micro-CT for quantifying the time-course and staining characteristics of *ex-vivo* animal organs treated with iodine- and gadolinium-based contrast agents

Juliana Martins de S. e Silva,[1*] Julian Utsch,[1] Melanie A. Kimm,[2] Sebastian Allner,[1] Michael F. Epple,[1] Klaus Achterhold, [1] Franz Pfeiffer[1,2,3]

[1] Chair of Biomedical Physics, Department of Physics and Munich School of BioEngineering, Technical University of Munich, 85748 Garching, Germany.

[2] Department of Diagnostic and Interventional Radiology, Klinikum rechts der Isar, Technical University of Munich, 81675 München, Germany.

[3] Institute for Advanced Study, Technical University of Munich, 85748 Garching, Germany.

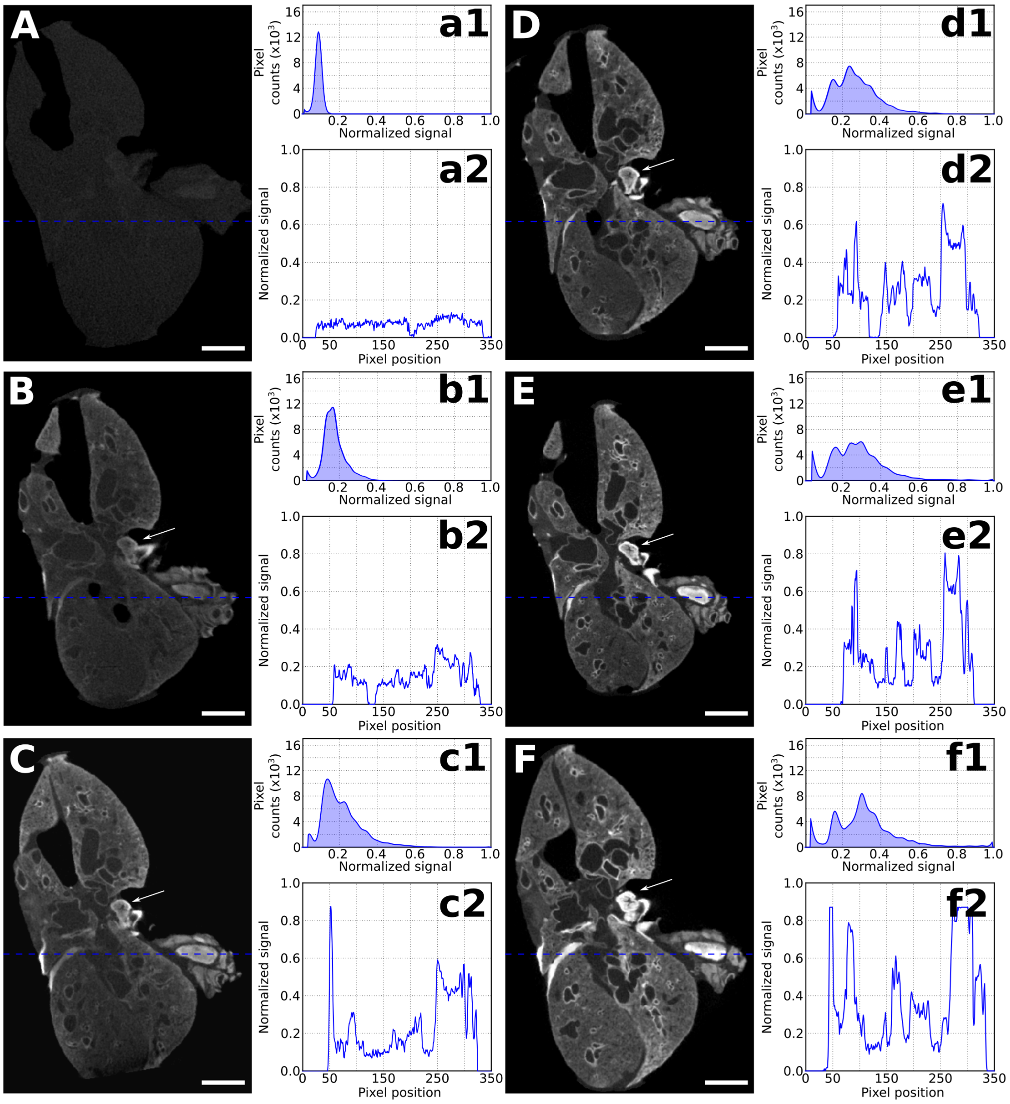

Figure S1. Comparison between X-ray micro-CT images (40 kVp) of non-stained and I2E-stained lungs with increasing staining times. Tomographic slices of the lungs are presented for staining times equal to (A) 0 min, (B) 30 min, (C) 60 min, (D) 90 min, (E) 120 min and (F) 150 min. The corresponding histograms (a1 - f1) and line plots (a2 - f2) are shown at the right side of each corresponding tomogram. All gray values were normalized to the maximum signal presented in Fig. 2 F. White arrows indicate the esophagus. Scale bar: 1000 μm.

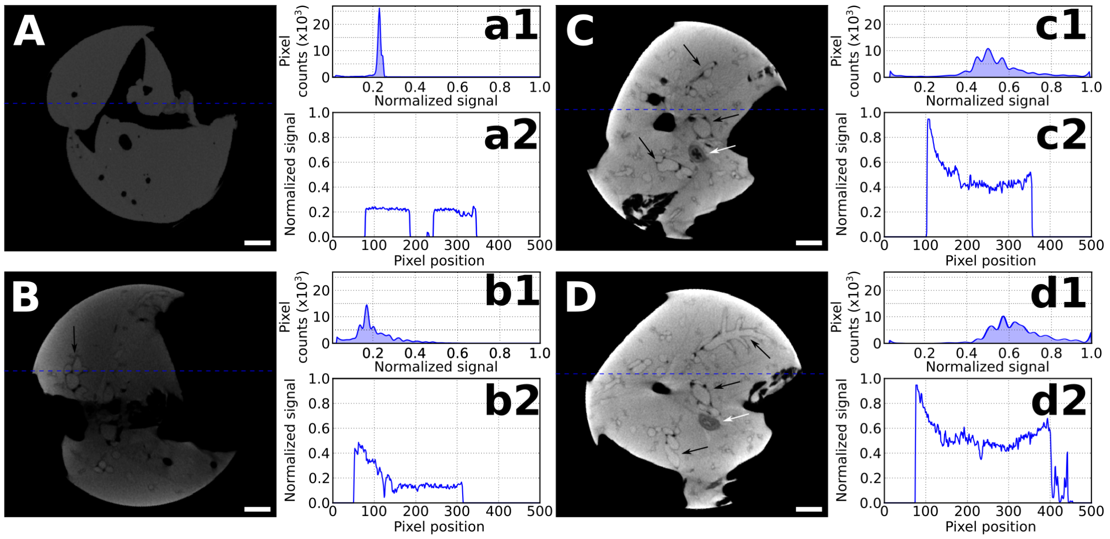

Figure S2. Comparison between dual-energy X-ray micro-CT images (70 kVp) of non-stained and Gadovist-stained lungs with increasing staining times. Tomographic slices of the lungs are presented for staining times equal to (A) 0 min, (B) 60 min, (C) 120 min and (D) 180 min. The corresponding histograms (a1 - d1) and line plots (a2 - d2) are shown at the right side of each corresponding tomogram. All gray values were normalized to the maximum signal presented in Fig. 2 F. White arrows indicate the esophagus and black arrows indicate some airways where the contrast agent has accumulated. Scale bar: 1000 µm.

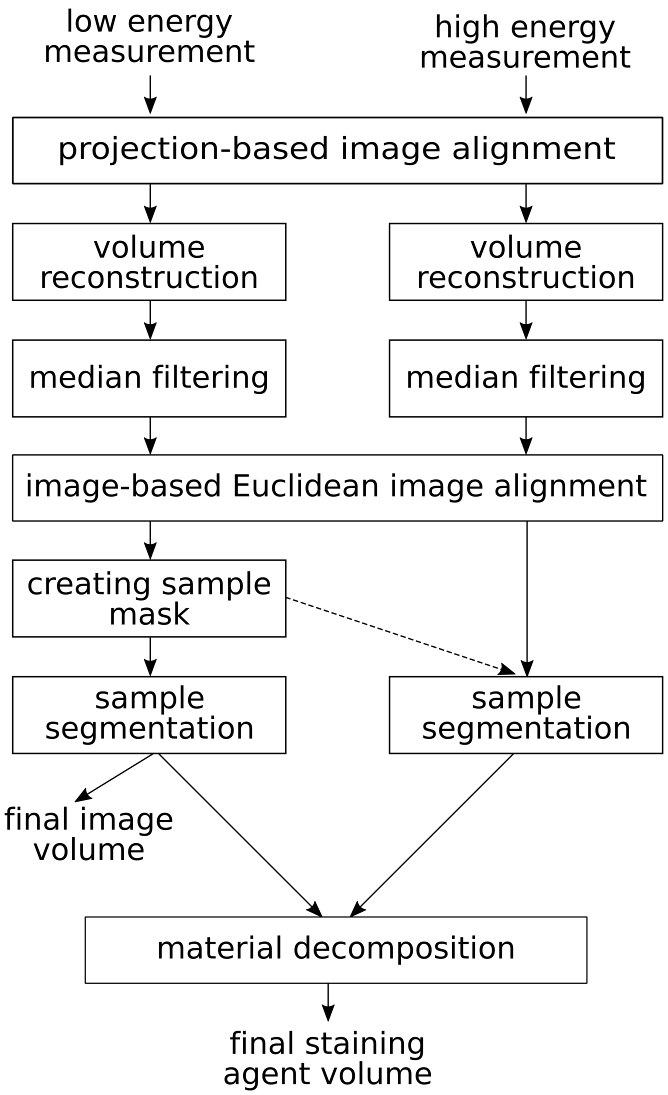

Figure S3. Dual-energy decomposition flow chart. Processing was done in Avizo Fire 8.1 and Python. Projections of the two measurements were aligned to each other by using a cross correlation algorithm in Python. The volumes were reconstructed and filtered for noise reduction independently from each other. Subsequently, the volumes were registered to each other by using Avizo's Image Registration Wizard based on an Euclidean metric. Image registration in both projection and image domain assures greatest precision. The sample mask was created manually and with linear interpolation and applied to both volumes, whereby the low energy one was used for the images presented in Fig. 2, 3, S2 and S3. Afterwards material decomposition was performed in Python.

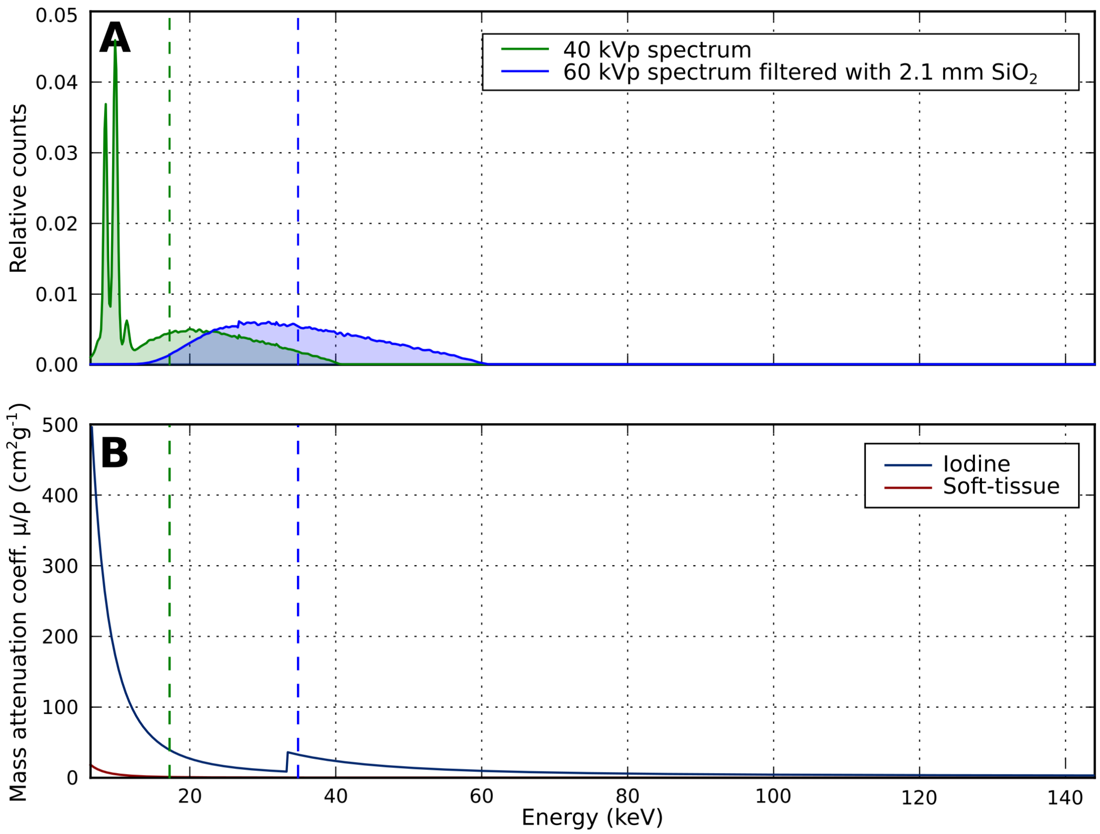

Figure S4. X-ray emission spectrum and energy attenuation curves of soft-tissue and iodine. (A) For the samples stained with I2E, the low energy (LE) measurement was performed at 40.2 keV (with an effective energy of $E_{eff,LE}$, centered at 17.2 keV), the high energy was 60.2 keV (with an effective energy of $E_{eff,HE}$ centered at 34.8 keV). The photon counts per energy were normalized to the intensity of the whole spectrum. The spectrum was measured with an Amptek X-123CdTe Spectrometer, and the SiO_2 filter material was simulated with values provided by the National Institute of Standards and Technology (NIST). The density was assumed as 2.6 g cm^{-3}. (B) The X-ray attenuation K-edge of iodine at 33.2 keV defined the choice of the individual energies used in this work. The attenuation curve data was provided by NIST.

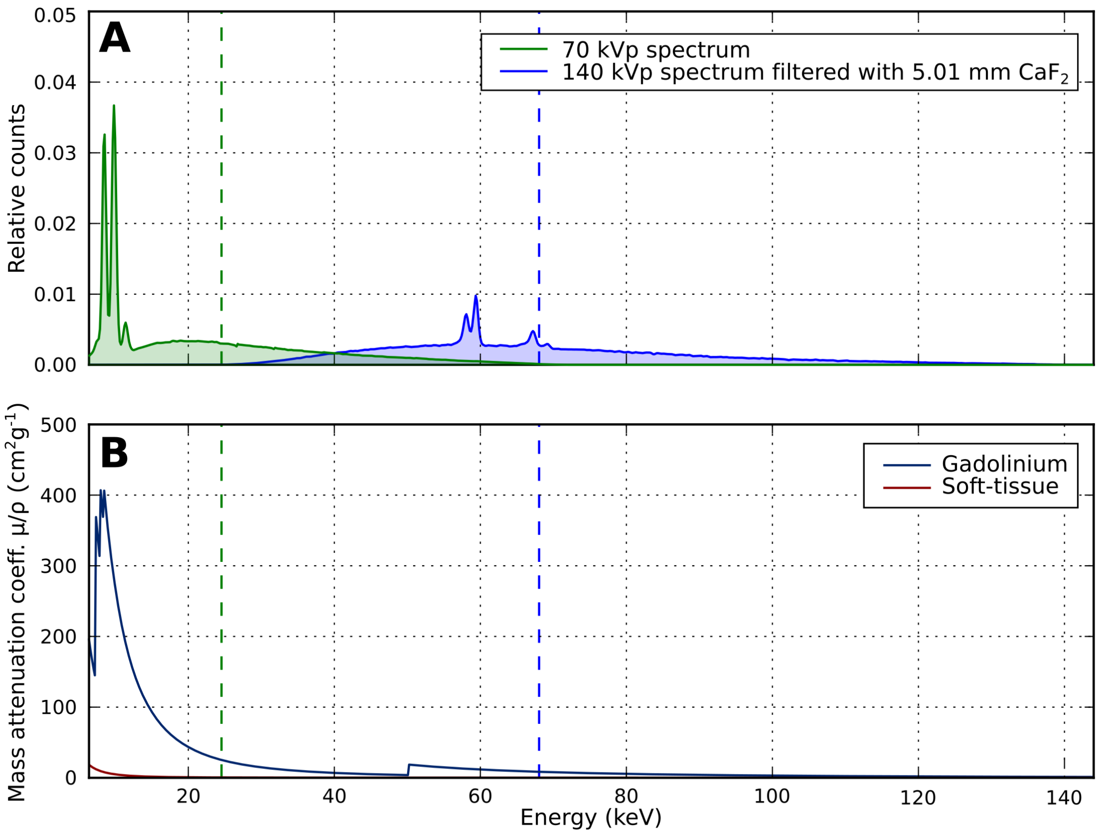

Figure S5. X-ray emission spectrum and energy attenuation curves of soft-tissue and gadolinium. (A) For the samples stained with Gadovist, the low energy (LE) measurement was performed at 70.1 keV (with an effective energy of $E_{eff,LE}$, centered at 22.1 keV), the high energy was 140.0 keV (with an effective energy of $E_{eff,HE}$ centered at 68.0 keV). The photon counts per energy were normalized to the intensity of the whole spectrum. The spectrum was measured with an Amptek X-123CdTe Spectrometer, the CaF_2 filter material was simulated with values provided by the National Institute of Standards and Technology (NIST). The attenuation curve data in (B) where provided by NIST and visualize the X-ray attenuation related to the emission spectrum.

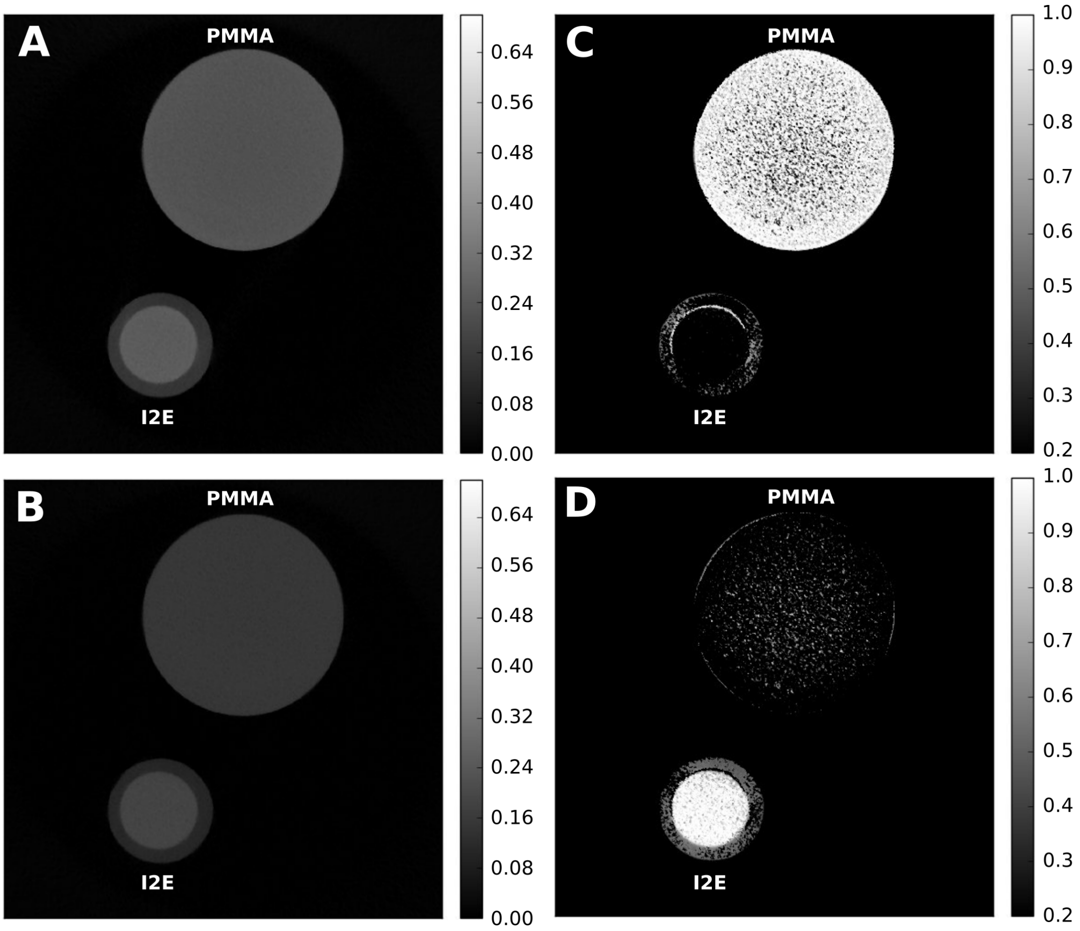

Figure S6. Measurement of attenuation of I2E solution and PMMA from calibration phantoms. (A) Low-energy and (B) high-energy scans of the phantom, (C) material decomposition based on PMMA and (D) material decomposition based on iodine (0.5 % I_2 in ethanol, 2 x 10^{-2} mol L^{-1} I_2).

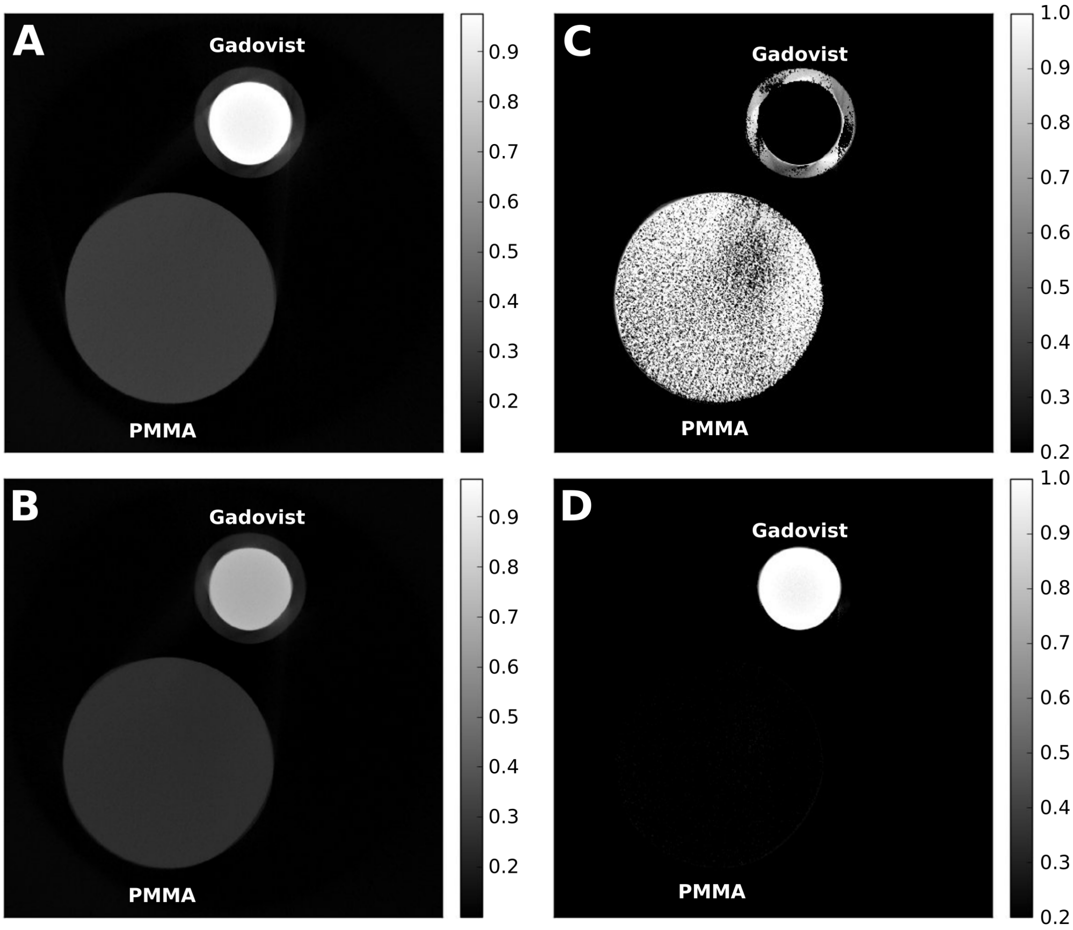

Figure S7. Attenuation measurement of Gadovist solution and PMMA from calibration phantoms. (A) Low-energy and (B) high-energy scans of the phantom, (C) material decomposition based on PMMA and (D) material decomposition based on gadolinium (pure Gadovist™, 1 mol L^{-1} Gd^{3+}).

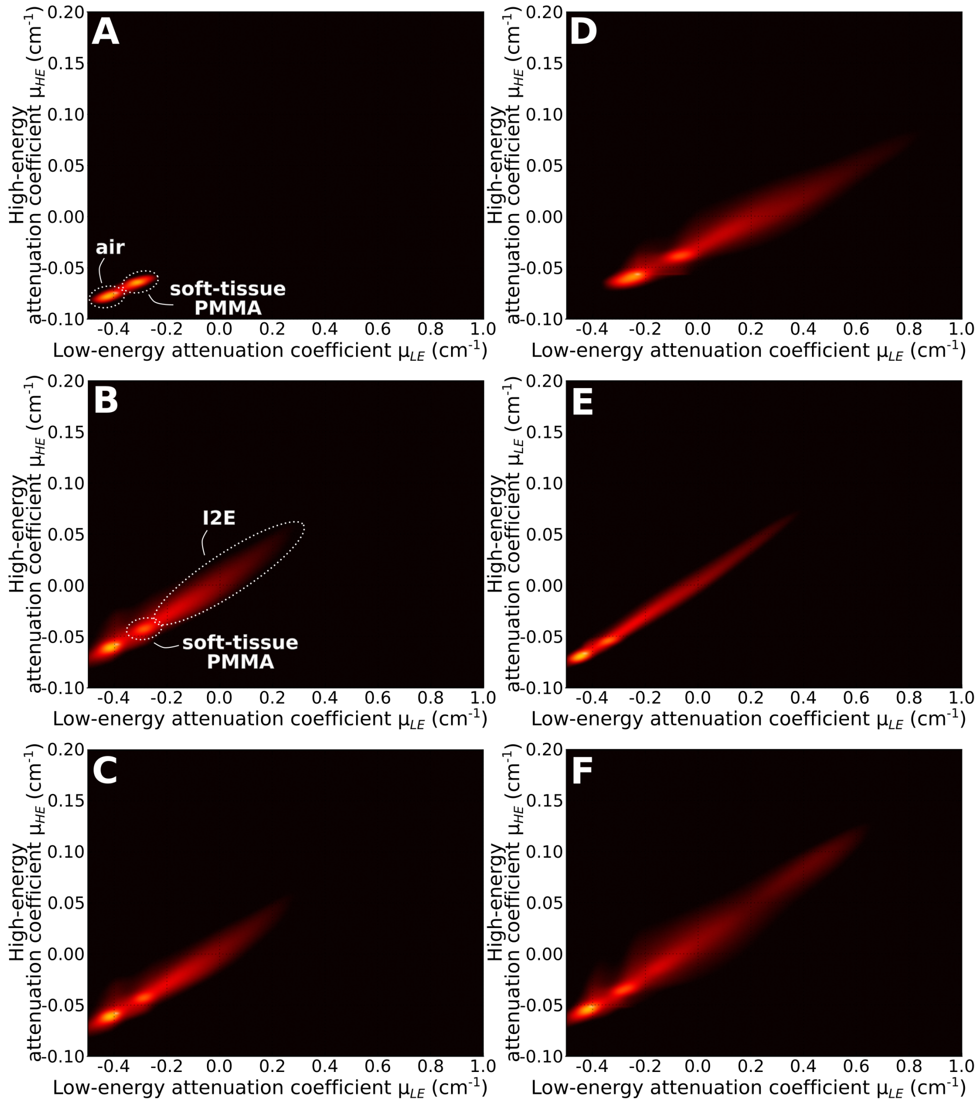

Figure S8. Correlation histograms for I2E-stained samples. The high-energy scans were performed with a 60.2 kV and a 2100 μm SiO_2 filter and the low-energy scans at 40.2 kV without filter, for (A) the unstained sample and the samples treated with I2E with staining times equal to (B) 30 min, (C) 60 min, (D) 90 min, (E) 120 min and (F) 150 min. Negative values result from X-ray scattering.

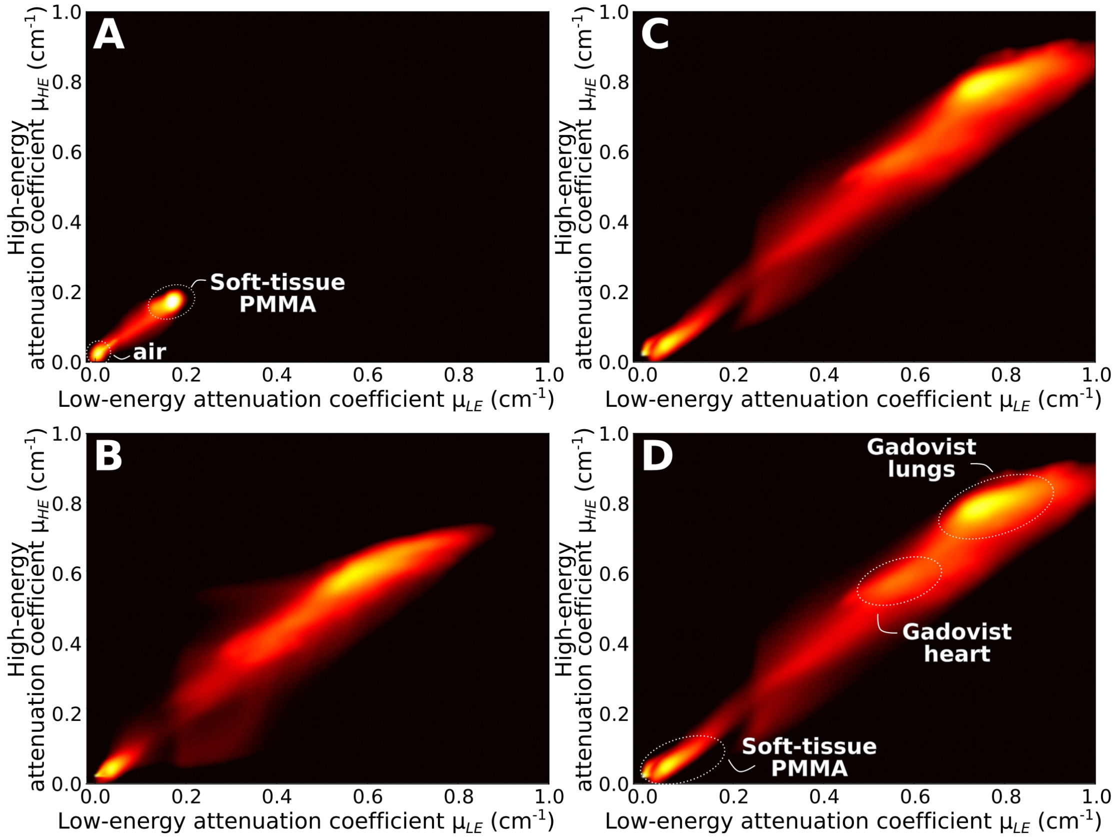

Figure S9. Correlation histograms for Gadovist-stained samples. The high-energy scans were performed with a 140.0 kV and a 5010 µm CaF_2 filter and the low-energy scans at 70.1 kV without filter, for (A) the unstained sample and the samples treated with Gadovist with staining times equal to (B) 60 min, (C) 120 min and (D) 180 min. Negative values result from X-ray scattering.

Table S1. Scanning parameters used in micro-CT measurements. Parameters used to perform low- and high-energy measurements of samples stained with iodine staining agent or with gadolinium-based contrast medium.

	Iodine	Gadolinium
Acceleration Voltage	40.2 kV/60.2 kV	70.1 kV/140.0 kV
Source Current	24.6 µA/16.3 µA	70.8 µA/35.7 µA
Filter Material	None/2100 µm SiO_2	None/5010 µm CaF_2
Camera Binning Factor	2	2
Exposure Time	3 s	1 s
Source-Sample-Distance	32.0 mm	40.0 mm
Sample-Detector-Distance	100.0 mm	90.0 mm
Pixel Size	16.5 µm x 16.5 µm	20.9 µm x 20.9 µm
Optical Magnification	0.39x	0.39x
No. of projections	1601	1601

Table S2. Effective attenuation coefficients determined by calibration measurement. Coefficients were measured on calibration materials with the same parameters as presented in Table S1.

Attenuation coefficient	Iodine [1/cm]	Gadolinium [1/cm]
$\mu_{stain,LE}$	0.253	0.685
$\mu_{stain,HE}$	0.189	0.486
$\mu_{PMMA,LE}$	0.228	0.166
$\mu_{PMMA,HE}$	0.154	0.127
$\mu_{air,LE}$	0.020	0.0007
$\mu_{air,HE}$	0.013	0.0002
